# The Relationship between Social Support and Mental Health Problems of Peri- and Postmenopausal Women during the SARS-CoV-2 Pandemic

**DOI:** 10.3390/ijerph20032501

**Published:** 2023-01-31

**Authors:** Anna Maria Cybulska, Katarzyna Głębicka, Marzanna Stanisławska, Aneta Cymbaluk-Płoska, Elżbieta Grochans, Kamila Rachubińska

**Affiliations:** 1 Department of Nursing, Faculty of Health Sciences, Pomeranian Medical University in Szczecin, 48 Żołnierska St., 71-210 Szczecin, Poland; 2 Department of Psychiatry, Pomeranian Medical University, 71-210 Szczecin, Poland; 3 Department of Gynecological Surgery and Gynecological Oncology of Adults and Adolescents, Pomeranian Medical University in Szczecin, 70-111 Szczecin, Poland

**Keywords:** anxiety, COVID-19, depression, menopause, mental health, social support

## Abstract

The COVID-19 pandemic affects women’s mental health, because they are more predisposed to vulnerabilities and adverse impacts. Therefore, is important to find strategies for preventing and treating these mental health consequences in the female population. The main purposes of our study were to determine the level of social support received by peri- and postmenopausal women during the SARS-CoV-2 pandemic, as well as factors related to this level with reference to health status and sociodemographic variables. A total of 218 women in peri- and postmenopausal status participated in the study. The study assessed depression (Beck Depression Inventory), anxiety (the Spielberg State-Trait Anxiety Scale), climacteric symptoms (the Blatt–Kupperman Index), social support (the Inventory of Social Supportive Behaviors). The majority of the respondents had a moderate level of anxiety as a state (40.8%), a low level of anxiety as a trait (51.4%), no depressive symptoms (75.2%) and no climacteric symptoms (52.3%). Age was found to significantly correlate with anxiety as a state (*p* = 0.036). The anxiety as state was significantly stronger in people with higher education than in people with secondary education (*p* = 0.019). Professionally inactive women had more emotional (*p* = 0.05) and appraisal (*p* = 0.014) support than women who work. The analysis demonstrated no statistically significant correlation between social support and depression, anxiety or climacteric symptoms (*p* > 0.05). The majority of peri- and postmenopausal women had no depressive symptoms and/or anxiety symptoms. Professionally inactive women had more emotional and appraisal support than women who work. The analysis demonstrated no statistically significant correlation between social support and depression, anxiety or climacteric symptoms.

## 1. Introduction

The COVID-19 pandemic, caused by the SARS-CoV-2 coronavirus, has been declared an international public health emergency. The World Health Organization (WHO) has expressed concern about the mental health, psychosocial and socio-economic consequences of the COVID-19 pandemic [1].

Already, at the beginning of the pandemic, it was observed that isolation or quarantine significantly affected the usual activities or livelihood of many people, which could result in an increase in the level of anxiety, depression, insomnia, alcohol or drug abuse, self-mutilation or even suicide [2]. The outburst of the COVID-19 pandemic and the following strict restrictions implemented by governments resulted in many dire consequences for the whole society. The most common symptoms were loneliness, increased anxiety and financial hardships caused by rapid halt of the economy. The global pandemic was a shock for many, and there was a lack of effective methods of coping with stress on such a scale.

This led to a further emotional burden, and thus to an increase in anxiety, sorrow or depressive symptoms. In most cases, people instinctively adapted to new conditions, but there were still many who needed help from a professional psychologist because long exposure to lockdown restrictions could lead to serious mental problems [3,4].

According to the WHO definition, mental health is a state of well-being in which an individual realizes his/her abilities, is able to cope with various life situations, and is able to participate in social life and work productively. Effective functioning in society and the foundation of well-being is a pillar of mental health, which means more than the absence of mental disorders [5]. Furthermore, mental health is considered as the most important condition for a good life quality. Stressful events, on the other hand, are strong adverse environmental factors may predispose to mental disorders [6].

Due to experiencing negative emotions during the COVID-19 pandemic, the National Health Commission has issued guidelines promoting psychological interventions aimed at civilians, medical staff and patients during the COVID-19 pandemic [7].

Social support (SS) is a necessary buffer of stressful life events, which is defined as resources provided by other people [8], which may be emotional, tangible, informative or evaluative [9]. In addition, it is verbal and non-verbal communication between recipients and service providers that reduces uncertainty about the situation, in relation to each other and relationships to strengthen the perception of personal control over one’s life [10]. The right amount of social support significantly improves mental health and relieves symptoms of depression, lowers the level of anxiety, improves self-efficacy and prevents loneliness [11]. Support networks develop throughout life and exist even when activation is not required. The most important sources of support are those natural, such as family, friends, relatives or social groups to which an individual functioning in a social environment belongs [12]. In addition, many scientists report that perceived support, which refers to the subjective sense of the potential availability of support, is a better predictor of well-being, coping with stress and health compared to the support received [13]. The availability of social support is a particularly important variable in the context of social and cultural determinants of the quality of life of women in the perimenopausal period [14].

A special period in the life of every woman is menopause, defined as: “The permanent cessation of menstruation due to the loss of ovarian follicle function. Biologically, menopause means a loss of fertility for a woman and is a natural physiological process that usually occurs between the ages of 45 and 55” [15]. Menopause is an important event in a woman’s life and is associated with symptoms such as hot flashes, night sweats, palpitations, mood swings, insomnia, anxiety, depression, attention deficit disorder, nervousness, headaches, mood swings, dysphoria, tension and tearfulness [16]. Hormonal changes taking place in a woman’s body significantly affect everyday functioning; moreover, there are many controversies regarding the role of menopause in the development of depression and anxiety [4]. It is worth noting that although there is an increased risk of clinical and subclinical depression during the period of reduced estrogen levels, its occurrence should not be directly attributed to menopause, but to various factors, including neurotransmitters, sociodemographic variables [17], psychosocial variables [18], personality traits [19] or genome [20,21].

Women in the perimenopausal period are particularly vulnerable to well-being disorders, especially during the COVID-19 pandemic. Both changes occurring in connection with menopause, as well as psychological or socio-economic consequences resulting from the duration of the COVID-19 pandemic, may negatively affect women’s mental health [5].

The aim of our study was to assess the mental state and social support of peri- and postmenopausal women during the SARS-CoV-2 pandemic. Moreover, the aim of this study was to assess whether sociodemographic variables (age, education, place of residence, marital status) and psychological distress of women (depression and anxiety) have an impact on the support received during the SARS-CoV-2 pandemic.

## 2. Materials and Methods

### 2.1. Settings and Design

The research was conducted from August to October 2021 among peri- and post-menopausal women in Zachodniopomorskie voivodeships (Poland). The inclusion criteria were female sex, the age of 41–75 years, no clinically confirmed mental disease and informed written consent to participate in the study. The exclusion criteria were: history of psychiatric treatment, no consent to participate in the study, age <41.

The size of the study sample was established on the basis of statistical data concerning the size of the 41–75 years old female population in the West Pomeranian Voivodeship in 2021. The confidence level was set at 95%, the maximum error at 7%, and the estimated fraction size at 0.5. The total number of women qualified for the study was 295.

A total of 295 peri- and post-menopausal women were invited to participate in the survey. Only 218 women correctly completed the surveys (completion rate: 74%). The majority of the respondents were postmenopausal women (55%).

The respondents were divided into two groups with regard to their menopausal status defined as [22]:

- Perimenopause―the time immediately before menopause with the symptoms of the coming menopause (when endocrine, biological and clinical features of the coming menopause begin);
- Postmenopause―the last menstruation at least 12 months before the study.

Recruitment of participants was carried out by means of information posters hung in public places and advertisements in local newspapers.

Respondents who met the inclusion criteria received general information regarding the course and purpose of the study, as well as instructions on completing the questionnaires. After giving consent to participate in the study, women received a questionnaire form. The respondents were informed that the study is entirely voluntary and anonymous as well as that the results obtained will be used for scientific purposes.

### 2.2. Ethical Considerations

The Bioethics Committee of the Pomeranian Medical University in Szczecin Approved this study ((KB-0012/46/01/2013). Approval number 6). This study was conducted as per the Declaration of Helsinki agreement. All participants were verbally informed about the study, and their consent was obtained.

### 2.3. Research Instruments

The factors influencing peri- and postmenopausal women’s mental health during the COVID-19 pandemic were determined using the following standardized survey instruments:

- Beck Depression Inventory (BDI) is a 21-question research instrument for measuring the severity of depression. There are four possible answers to each question, with the intensity of each response ranging from 0 (the least severe symptom) to 3 (the most severe symptom). The total score reflects the degree of depressive symptoms. No depression (0–11 points), mild depression (12–19 points), moderate depression (20–25 points), and severe depression (26–plus points) were the four score ranges used in the study [23]. Additionally, we divided patients into those who had depression (12 points or more) and those who did not have it (less than 12 points). The BDI’s Cronbach’s alpha was 0.89 [24].
- The Spielberg State-Trait Anxiety Scale (STAI) is a tool used in research to assess both trait and state anxiety. No anxiety (≤20), mild anxiety (21–39), moderate anxiety (40–59), and severe anxiety (60–80) are the four categories that make up the STAI questionnaire score, which ranges from 20 to 80 points. The range of Cronbach’s alphas for trait anxiety and state anxiety respectively was 0.86 to 0.92 and 0.83 to 0.92 [25].
- The Blatt–Kupperman Index (BKMI) evaluates climacteric symptoms using an 11-item questionnaire. The questionnaire asks about both physical and psychological symptoms, such as fatigue, anxiety, and melancholy. The physical symptoms listed include sweating/hot flushes, palpitations, vertigo, headaches, paresthesia, and arthralgia and myalgia. These complaints are rated from 0 to 3 in terms of their seriousness. The sum of all the items determines the final score. The study adopted the following score ranges: 0–16 = no symptoms, 17–25 = mild symptoms, 26–30 = moderate symptoms, and ≥31 = severe symptoms [26].
- The Inventory of Social Supportive Behaviors (ISSB) is a 40-item self-report questionnaire that was created to gauge how frequently people received different types of assistance in the month prior. The tool conceptualizes social support as including both concrete forms of assistance, like the provision of goods and services, and intangible forms of assistance, like advice and expressions of respect. On 5-point Likert scales (1 = not at all, 2 = once or twice, 3 = roughly once a week, 4 = several times a week, and 5 = roughly every day), subjects are asked to rate the frequency of each item. The ISSB divides support into four categories: instrumental support, information support, emotional support, and appraisal support. The ISSB’s Cronbach’s alpha was 0.90 [27,28].

The demographic information (age, marital status, place of residence, education, and employment status), the medical information (menstruation, menopausal syndromes), the history of exposure to COVID-19, and any other pertinent information regarding COVID-19 were all gathered using the author’s questionnaire.

### 2.4. Statistical Analysis

The analysis of quantitative variables (expressed numerically) was performed by calculating the mean, standard deviation, median, quartiles, and minimum and maximum values. Comparison of quantitative variables in the two groups was performed using the Mann-Whitney U test. Correlations between quantitative variables were analyzed using Spearman’s correlation coefficient. The Kruskal-Wallis test and the Dunn test were used in the study.

All calculations were performed using R version 4.1.2. (RStudio, Boston, MA, USA). The level of statistical significance was set at *p* < 0.05 [29].

## 3. Results

### 3.1. Characteristics of the Respondents

The study sample consisted of 218 women who correctly completed the questionnaires. The mean age was 53 years (SD = 6.7). The majority of the respondents were female in a formal relationship (57.8%), achieved higher education (54.13%), living in a city of more than 100 thousand residents (59.17%) and professionally active (83.94%). Of the 218 surveyed women, 55.1% were respondents who had their last menstrual period at least 12 months before the study. The age of the last menstruation in postmenopausal women averaged 49.39 years (SD = 3.71).

A total of 41.3% of the respondents get over COVID-19 but 92.7% were not hospitalized for COVID-19. 58.26% felt fear of COVID-19 and 58.3% of the subjects were in quarantine due to COVID-19.

Among the surveyed women, 62.4% had a person in their immediate family who was ill with COVID-19. In addition, 22.94% of respondents lost a loved one during the SARS-CoV-2 pandemic. The vast majority of respondents (78%) had limited contact with their loved ones and were vaccinated against COVID-19 (76%).

### 3.2. The Severity of Depression, Anxiety, Climacteric Symptoms and Social Support among the Peri- and Postmenopausal Women during the SARS-CoV-2 Pandemic

Analysis was performed on depressive symptoms (according to the BDI), climacteric symptoms (according to the BKMI), anxiety (according to the STAI), and social support (according to the MSPSS) among peri- and postmenopausal women of the West Pomeranian Voivodeship during the SARS-CoV-2 pandemic.

A total of 75.2% of the subjects had no depressive symptoms, while 13.8% showed mild, 7.3% moderate, 3.7% severe symptoms of depression according to the BDI.

The majority of the respondents had moderate level of anxiety as a state (40.8%) and low level of anxiety as a trait (51.4%).

The BKMI diagnosed severe climacteric symptoms in only 14.2 % of the women analyzed, moderate symptoms in 7.8 % of the women and minor symptoms in 25.7% of the women, and 52.3 % of the women had no symptoms at all.

The analysis of the results obtained from the ISSB showed that the average score for the emotional support subscale was 32.14 points, for information support 46.77 points, for instrumental support 58.83 points, and for appraisal support 17.44 points. This means that the respondents receive emotional, information and instrumental support several times a week. In turn, they receive appraisal support once a week (Table 1).

### 3.3. Analysis of the Relationship between Sociodemographic Variables (Age, Education, Place of Residence, Marital Status) and Medical Variables (Menopausal Status, Get over COVID-19) on the Severity of Depression, Anxiety, Climacteric Symptoms and Social Support among the Peri- and Postmenopausal Women during the SARS-CoV-2 Pandemic

This study analyzed the influence of selected sociodemographic variables (age, education, place of residence, marital status) severity of depression, anxiety, climacteric symptoms and social support among the peri- and postmenopausal women during the SARS-CoV-2 pandemic.

It was found that age significantly correlates with anxiety as a state (*p* = 0.036 r = −0.142). There were no statistically significant correlations between age and anxiety as a trait and no statistically significant correlations between age and severity of depression (Table 2). The analysis of the impact of other sociodemographic variables (marital status, place of residence, education and professional activity) on the severity of depression among peri- and postmenopausal women during the SARS-CoV-2 pandemic showed no statistically significant correlations (Table S1).

The anxiety as state was significantly stronger in people with higher education than in people with secondary education.

Analysis of the data did not demonstrate the difference in education on anxiety as a trait. Analysis of the influence of other sociodemographic variables (marital status, place of residence, professionally active) on the severity of anxiety among the peri- and postmenopausal women during the SARS-CoV-2 pandemic did not reveal any statistically significant difference (*p* > 0.05) (Table 3).

The analysis demonstrated no statistically significant relationships between climacteric syndrome according to the BKMI and sociodemographic variables (age, marital status, place of residence, education, professionally active) among the peri- and postmenopausal women during the SARS-CoV-2 pandemic (Table S1).

There were no statistically significant correlations between age and social support according to ISSB (Table 2). Data analysis showed statistically significant differences in emotional and value support according to the ISSB, taking into account the professional activity of the respondents. Professionally inactive women showed higher levels of emotional support (*p* = 0.05) and evaluative support (*p* = 0.014). An analysis of the influence of other sociodemographic variables (marital status, place of residence, education) on the social support among the peri- and postmenopausal women during the SARS-CoV-2 pandemic did not reveal any statistically significant difference (*p* > 0.05) (Table S2).

The anxiety as state was significantly stronger in perimenopausal women than in postmenopausal women (*p* = 0.028). The analysis demonstrated no statistically significant influence of menstruation on anxiety as a trait. The anxiety as state (*p* = 0.016) and a trait (*p* = 0.011) were significantly stronger in women who get over COVID-19 (Table 4). Menopausal status and get over of COVID-19 were not a statistically significant contributors to depressive symptoms in women during the SARS-CoV-2 pandemic (Table 3).

The analysis demonstrated no statistically significant difference between climacteric syndrome according to the BKMI, depression according of the BDI or social support according ISSB and medical variables (menopausal status, get over COVID-19) among the peri- and postmenopausal women during the SARS-CoV-2 pandemic (*p* > 0.05) (Table S3).

The climacteric symptoms according of BKMI was found to significantly correlate with anxiety as a state (*p* < 0.001, r = 0.34) and anxiety as a trait (*p* < 0.001, r = 0.315) (Table 5). This means that the stronger the accident symptoms, the greater the severity of anxiety.

The analysis demonstrated statistically positive significant correlation between climacteric syndrome according to the BKMI and depression according of the BDI among the peri- and postmenopausal women during the SARS-CoV-2 pandemic. It was shown that the greater the accident symptoms, the stronger the intensity of depressiveness (Table 5).

### 3.4. Analysis of the Correlations between Social Support and Severity of Depression, Anxiety, Climacteric Symptoms among the Peri- and Postmenopausal women during the SARS-CoV-2 Pandemic

The analysis demonstrated no statistically significant correlation between social support according to the ISSB and depression according of the BDI among the peri- and postmenopausal women during the SARS-CoV-2 pandemic. There were no statistically significant correlations between social support according to ISSB and anxiety according of STAI. The social support was not found to significantly correlate with climacteric symptoms according of BKMI (Table 6).

## 4. Discussion

### 4.1. The Severity of Depression, Anxiety, Climacteric Symptoms and Social Support among the Peri- and Postmenopausal Women during the SARS-CoV-2 Pandemic

The COVID-19 pandemic has significantly affected the psychological well-being of people around the world. It is worth noting that mental health issues during the COVID-19 epidemic are related to various biopsychosocial and COVID-19-related factors [30]. Women in the perimenopausal period were particularly vulnerable to disturbances of well-being in this period. Menopause is associated with a decrease in estrogen levels, which in turn contributes to the occurrence of depressive symptoms and mood disorders. The authors of the presented study assessed the mental state and social support of women in the perimenopausal and postmenopausal periods, and also attempted to search for significant predictors among the sociodemographic variables and psychological distress of women in the perimenopausal period during the COVID-19 pandemic.

Our own research showed that the vast majority of respondents had no depressive symptoms and had no climacteric symptoms. Moreover, the majority of the respondents had a moderate level of anxiety as a state and a low level of anxiety as a trait. The women received emotional, informational, and instrumental support several times a week. In turn, they receive appraisal support once a week.

On the other hand, research by Cybulska et al. [4] showed that during the pandemic, women experienced moderate to severe depression. Wang et al. [31] or Qui et al. [32] also confirm the significant impact of COVID-19 on the deterioration of the mental state of respondents, especially among women, where the impact of the epidemic on mental functioning was more pronounced. Metaanaliza Luo et al. [33] showed that one-third of adults in the general population suffered from anxiety (33% (28–38%)) or depression (28% (23–32%)), on the other hand Wang et al. [34] found that the prevalence of anxiety was (33% (28–39%)) and depression (30% (26–36%)). Babicki et al. [35] observed that the vast majority of respondents (75%) reported anxiety symptoms of varying severity. Similar results were obtained by Milton et al. [36].

Furthemore, Research by Wu et al. [37] found that COVID-19 survivors with higher perceived life threat, less emotional support, less severity of illness on admission, and longer hospital stay were associated with a greater severity of PTSD, anxiety and depression symptoms.

### 4.2. Sociodemographic Variables (Age, Education, Place of Residence, Marital Status) and Medical Variables Relationship Analysis (Menopausal Status, Get over COVID-19) on the Severity of Depression, Anxiety, Climacteric Symptoms and Social Support among the Peri- and Postmenopausal Women during the SARS-CoV-2 Pandemic

Our own research showed that sociodemographic and medical variables had no significant effect on the occurrence of depressiveness.

An analysis of the literature proves that high levels of anxiety, stress and depressive symptoms mainly concern young people, women and people with children [35,36,37,38,39,40,41,42,43]. This is in line with the reports of Cybulska et al. [4], which confirm that gender significantly differentiated the study group in terms of both anxiety and depressive symptoms. Women are more likely to experience depression than men, and declared significantly more concerns about everyday life during the pandemic than men. Additionally, Jasik et al. [44] and Firat et al. [45] found that women showed significantly more depressive symptoms.

In turn, Ozamiz-Etxebarria et al. [46] notes that not only the symptoms of depression, but also anxiety or stress are more severe in women than in men.

Studies by Çalişkan et al. [47] showed that women are more prone to depression in the premenopausal and postmenopausal periods. Being single/widowed, low income, low social support and a poor quality of life are important risk factors that increase the incidence of depression. In addition, a weak negative correlation was found between depression and social support score.

A review of the literature indicates common risk factors for mental disorders, which included being, for example, a woman [48,49,50,51,52,53] and a person with lower socio-economic status [51,54,55,56,57,58,59]. Family support, on the other hand, was a protective factor against anxiety [58].

The Global Burden Disease of Study [60] confirms that anxiety and depression were much more common in women than in men. The reasons for the differences between the sexes are not well understood, possibly due to fluctuations in sex hormone levels or a drop in estrogen levels [61].

### 4.3. Analysis of the Correlations between Social Support and Severity of Depression, Anxiety, Climacteric Symptoms among the Peri- and Postmenopausal Women during the SARS-CoV-2 Pandemic

Many studies prove that support mitigates the negative health effects associated with stress [62], and a favorable environment reduces the symptoms of stress [63,64,65]. Our own research demonstrated no statistically significant correlation between social support according to the ISSB and depression according of the BDI among the peri- and postmenopausal women during the SARS-CoV-2 pandemic. There were no statistically significant correlations between social support according to ISSB and anxiety according of STAI.

Research by Arnot et al. [66] showed that increased social support is not associated with a reduction in menopausal symptoms, nor does it attenuate the negative relationship between stress and menopausal symptoms. Zhao et al. [64] showed that the symptoms of menopause differ in different periods of menopause. In addition, greater resilience and family support were significantly associated with fewer menopausal symptoms, which may be helpful to health care professionals in identifying these symptoms and seeking for appropriate preventive intervention.

Research by McLean et al. [67] showed that emotional and instrumental social support was significantly associated with perceived symptoms of stress, anxiety and depression. It was noted that greater use of emotional support was consistently associated with better outcomes, while the use of instrumental support was associated with poorer outcomes. For both men and women, emotional support was associated with fewer anxiety symptoms and was associated with less stress.

These results are consistent with global research on coping styles and mental health outcomes during the COVID-19 pandemic. Gurvich et al. [68] showed that the use of instrumental social support was associated with higher anxiety, although neither emotional nor instrumental support was associated with depression or stress. This is in line with research by Agha at al [69].

A review of the literature indicates that various types of support (family, emotional, etc.) reduce the number of menopausal symptoms [19,64,65].

Li et al. [70] found that the impact of sleep quality on subjective well-being was partly mediated by anxiety symptoms, and social support moderated the relationship between anxiety symptoms and subjective well-being among women in the perimenopausal period.

The pandemic caused by the SARS-CoV-2 virus causes fear and anxiety in the whole society, in particular in perimenopausal and postmenopausal women. The topic taken up in the work requires a lot of research to determine in detail the impact of individual characteristics on the psychosocial functioning of the respondents. It is worth emphasizing that emotional social support plays a protective role for psychological outcomes, while instrumental social support is associated with more intense perceived stress, anxiety and depressive symptoms during the COVID-19 pandemic [67]. The use of emotional support seems particularly important for women to help cope with distress during a public health emergency characterized by uncertainty. In addition, because informational social support may be ineffective in the case of increasing stress, anxiety and depression, some alternative solutions should be sought.

## 5. Limitations

The digressions presented in this research have defined certain limitations and implications for professional practice. There is a rich literature covering the impact of the COVID-19 pandemic on the mental health of the population (in which the incidence of depression, anxiety, the level of stress or the need for social support was assessed). However, studies rarely concern women in the perimenopausal period, who, due to hormonal changes, are particularly susceptible to mood changes, depression, anxiety or stress.

Unfortunately, our study has some limitations. First, the study used a self-report questionnaire. Respondents could respond to it in a socially acceptable way. However, the research assumption was based on trust and understanding of the questions by the respondents. In addition, the selection of respondents was random. 

In addition, we consider the small group of women surveyed as a limitation of the study, in which the menopausal status was determined on the basis of a questionnaire, and not sex hormone level tests.

Despite the limitations of our study, it is worth mentioning that its advantage was the individual approach to the study group. Activities that recognize women’s needs and aid in adjustment to the new reality must be included. Allowing for the potential use of psychological assistance seems crucial. Institutional preventive measures must also be taken to avoid psychological issues during and after the COVID-19 pandemic. Therefore, it is advisable to conduct further research and take preventive measures to protect the mental health of women in the perimenopausal period.

## 6. Conclusions

The majority of peri- and postmenopausal women had no depressive symptoms and/or anxiety and/or climacteric symptoms, whereas had social support especially emotional, informational and instrumental support. Professionally inactive women had more emotional and appraisal support than women who work. However, social support according to the ISSB did not affect depression, anxiety or climacteric symptoms among the peri- and postmenopausal women during the SARS-CoV-2 pandemic. Age and education were the variables that significantly affected the severity of anxiety as a state of the studied the peri- and postmenopausal women during the SARS-CoV-2 pandemic. Anxiety as a state was significantly stronger in people with higher education than in people with secondary education. Despite the significant impact of the COVID-19 pandemic on women’s mental health, there are few studies of perimenopausal women that have assessed the level of social support received by peri- and postmenopausal women during the SARS-CoV-2 pandemic and the factors associated with this level in relation to health status and sociodemographic variables. That is why it is crucial to conduct more research that will enable the adoption of preventative measures to shield women from the onset of depressive symptoms or an increase in anxiety. Additionally, it needs to be determined what kind of assistance enables women to maintain their good mental health.

## Figures and Tables

**Table 1:** Social support according to ISSB.

ISSB	M	SD	Average per Question	Me	Min–Max	Q1–Q3
emotional support	32.14	8.17	3.57	33	9–45	27–38
information support	46.77	11.50	3.90	50	12–60	40.25–56
instrumental support	58.83	10.85	4.20	62	16–70	52.55–67
appraisal support	17.44	5.14	3.49	18	5–25	14–21

M—mean; SD—standard deviation, Me—median, Min—minimum Max—maximum Q—quartile, ISSB—the inventory of social supportive behaviors.

**Table 2:** Influence of age on the occurrence of depression according to the BDI, anxiety according to the STAI, climacteric symptoms according to the BKMI and social support according to the ISSB.

Variables		Age	
		r	*p*
BDI		−0.095	0.164
STAI	STAI-I (state)	−0.142	0.036
	STAI-II (trait)	−0.107	0.115
BKMI		0.069	>0.05
ISSB	emotional support	0.021	0.759
	information support	0.086	0.206
	instrumental support	0.034	0.617
	appraisal support	0.113	0.097

BDI—Beck depression inventory; STAI—the Spielberg state-trait anxiety scale; BKMI—the Blatt–Kupperman index; ISSB—the inventory of social supportive behaviors; *p*—significance level.

**Table 3:** The anxiety (state and trait) of the respondents depending on socio demographic variables.

Variables		STAI-I (State)		STAI-II (Trait)	
		M	SD	M	SD
Marital status	Formal relationship (n = 126)	42.09	11.73	43.6	10.0
	Informal relationship (n = 24)	40.42	10.21	45.12	8.03
	Divorced (n = 31)	41.29	7.88	44.35	9.48
	Single (n = 17)	45.29	13.71	45.12	12.73
	Widowed (n = 20)	38.95	9.76	40.78	9.88
	*p* *	0.569		0.583	
Place of residence	Village (n = 34)	45.56	12.35	44.21	9.93
	City with up to 10,000 people (n = 15)	42.2	8.91	46.67	10.89
	City with 10,000–100,000 people (n = 40)	42	9.96	45.58	9.01
	City with over 100,000 people (n = 129)	40.62	11.2	42.7	10.07
	*p* *	0.2		0.295	
Education	Basic [A] (n = 11)	44.45	12.08	45.91	9.74
	Professional [B] (n = 24)	39.12	11.1	41.33	9.11
	Secondary [C] (n = 65)	38.75	10.87	43.4	10.21
	Higher [D] (n = 118)	43.69	10.78	44.21	10.01
	*p* ^	0.019	D > C *	0.553	
Professionally active	Yes (n = 183)	42.23	11.35	43.75	9.85
	No (n = 35)	39.26	9.4	43.66	10.61
	*p* ^&^	0.242		0.928	

* Kruskal-Wallis test; ^ *p*—Kruskal-Wallis test + post-hoc analysis (Dunn’s test); ^&^ Mann-Whitney test; *p*—significance level. STAI—the Spielberg state-trait anxiety scale.

**Table 4:** The anxiety (state and trait) of the respondents depending on menopausal status and get over COVID-19.

Variables		STAI-I (State)		STAI-II (Trait)	
		M	SD	M	SD
Menopausal status	Perimenopasal women (n = 98)	43.66	11.57	45.05	9.06
	Postmenopausal women (n = 120)	40.19	10.49	42.66	9.85
	*p* *	0.028		0.056	
Get over COVID-19	Yes (n = 90)	44.21	11.7	45.85	9.58
	No (n = 128)	40.02	10.35	42.25	9.97
	*p* *	0.016		0.011	

M—mean; SD—standard deviation, *p*—significance level, STAI—the Spielberg state-trait anxiety scale; * Mann-Whitney test.

**Table 5:** Correlations between climacteric symptoms and psychosocial functioning variables (depression according to the BDI, anxiety according to the STAI).

Variables		BKMI	
		r	*p*
BDI		0.328	<0.001
STAI	STAI-I (state)	0.34	<0.001
	STAI-II (trait)	0.315	<0.001

BDI—Beck depression inventory; STAI—the Spielberg state-trait anxiety scale; BKMI—the Blatt–Kupperman index; *p*—significance level.

**Table 6:** Correlations between social supports and occurrence of depression according to the BDI, anxiety according to the STAI, Climacteric symptoms according to the BKMI.

Variables		ISSB							
		Emotional Support		Information Support		Instrumental Support		Appraisal Support	
		r	*p*	r	*p*	r	*p*	r	*p*
BDI		0.06	0.382	−0.006	0.925	−0.023	0.733	0.094	0.167
STAI	STAI−I (state)	0.062	0.364	−0.055	0.422	0.069	0.312	0.079	0.243
	STAI−II (trait)	0.063	0.358	−0.018	0.788	−0.067	0.324	0.131	0.054
BKMI		0.026	0.7	−0.048	0.479	−0.039	0.571	0.054	0.423

BDI—Beck depression inventory; STAI—the Spielberg state-trait anxiety scale; BKMI—the Blatt–Kupperman index; *p*—significance level.

## Data Availability

Not applicable.

## Supplementary Materials

The following supporting information can be downloaded at: https://www.mdpi.com/article/10.3390/ijerph20032501/s1, Table S1: The level of depression according of BDI and climacterium symptoms according of BKMI of the respondents depending on socio demographic variables; Table S2: The social support according of ISSB of the respondents depending on socio demographic variables; Table S3: The level of depression according of BDI and climacterium symptoms according of BKMI of the respondents depending on medical variables; Table S4: The social support according of ISSB of the respondents depending on medical variables.
