# Supplementary material for: The Relationship between Social Support and Mental Health Problems of Peri- and Postmenopausal Women during the SARS-CoV-2 Pandemic
Source: Int J Environ Res Public Health. 2023 Jan 31;20(3):2501. doi: 10.3390/ijerph20032501 (PMC9916386; doi:10.3390/ijerph20032501)
Supplement: Supplementary file 1 [file ijerph-20-02501-s001.zip › ijerph-2111067-supplementary.pdf]

Table S1. The level of depression according of BDI and climacterium symptoms according of BKMI of the respondents depending on socio demographic variables

Variables		BDI		BKMI	
		**M**	**SD**	**M**	**SD**
**Marital status**	Formal relationship (n=126)	8.86	8.28	17.6	11.34
	Informal relationship (n=24)	9.96	8.45	15.33	10.62
	Divorced (n=31)	6.19	6.53	18	11.78
	Single (n=17)	10.65	10.76	10.94	8.21
	Widowed (n=20)	7.15	8.05	19.85	10.27
	p*	0.304		0.099	
**Place of residence**	Village (n=34)	11.68	9.72	18.85	10.88
	city with up to 10,000 people (n=15)	7.87	1	16.93	13.7
	city with 10,000–100,000 people (n=40)	8.32	8.47	18.05	10.78
	city with over 100,000 people (n=129)	7.93	7.88	16.35	11.03
	p*	0.141		0.553	
**Education**	Basic [A] (n=11)	13.27	10.18	14.64	11.29
	Professional [B] (n=24)	7.12	6.84	18.58	12.25
	Secondary [C] (n=65)	6.52	6.54	16.89	10.59
	Higher [D] (n=118)	9.58	8.96	17.13	11.26
	p*	0.063		0.855	
**Professionally active**	Yes (n=183)	8.8	8.53	17.32	10.41
	No (n=35)	7.43	6.95	15.89	14.43
	p[&]	0.473		0.228	

M—mean; SD—standard deviation, p—significance level, * Kruskal-Wallis test ^ & Mann-Whitney test, p—significance level BDI- Beck Depression Inventory; BKMI- The Blatt–Kupperman Index; p—significance level

Table S2. The social support according of ISSB of the respondents depending on socio demographic variables

Variables		ISSB							
		emotional support		information support		instrumental support		appraisal support	
		M	SD	M	SD	M	SD	M	SD
**Marital status**	Formal relationship (n=126)	31.95	8.38	46.82	11.61	59.41	10.35	17.42	5.16
	Informal relationship (n=24)	31.33	7.85	44.17	11.47	57.67	11.33	16.58	5.5
	Divorced (n=31)	30.94	7.83	45.35	12.08	55.55	12.39	17.52	4.63
	Single (n=17)	35.53	6.78	48.94	10.97	59.24	12.12	17.82	5.55
	Widowed (n=20)	33.3	8.34	49.95	10.34	61.35	9.48	18.1	5.39
	p*	0.311		0.304		0.236		0.865	
**Place of residence**	Village (n=34)	31.91	9.09	46.29	12.38	57.35	13.2	17.26	6.25
	city with up to 10.000 people (n=15)	35.53	8.19	50.67	10.28	61.13	7.99	18.6	5.64
	city with 10.000–100.000 people (n=40)	32.17	8.05	45.55	11.76	57.6	11.37	17.6	5
	city with over 100.000 people (n=129)	31.8	7.9	46.82	11.35	59.34	10.31	17.29	4.85
	p*	0.505		0.418		0.843		0.812	
**Education**	Basic [A] (n=11)	34.09	11.24	47.36	14.17	54.09	18.69	18.09	7.79
	Professional [B] (n=24)	33.21	6.73	46.54	10.02	59.42	9.08	17.17	4.82
	Secondary [C] (n=65)	32.83	8.32	47.82	11.06	59.38	10.48	17.92	4.89
	Higher [D] (n=118)	31.36	7.99	46.19	11.86	58.86±10.48	10.48	17.16	5.1
	p*	0.297		0.722		0.986		0.497	
**Professionally active**	Yes (n=183)	31.65	8.38	46.15	11.9	58.14	11.39	17.07	5.24
	No (n=35)	34.71	6.23	50.03	8.6	62.46	6.41	19.37	4.16
	p&	**0.05**		0.109		0.073		**0.014**	

M—mean; SD—standard deviation. p—significance level. * Kruskal-Wallis test ^ & Mann-Whitney test, p—significance level , ISSB- The Inventory of Social Supportive Behaviors

Table S3. The level of depression according of BDI and climacterium symptoms according of BKMI of the respondents depending on medical variables

Variables		BDI		BKMI	
		**M**	**SD**	**M**	**SD**
**Menopausal status**	Perimenopasal women (n=98)	9.46	8.95	15.6±	10.62
	Postmenopausal women (n=120)	7.87	7.68	18.3	11.42
	p*	0.264		0.077	
**Get over COVID-19**	Yes (n=90)	9.9	9.02	18.94	11.82
	No (n=128)	7.66	7.65	15.79	10.45
	p*	0.08		0.069	

M—mean; SD—standard deviation. p—significance level. Mann-Whitney test. BDI- Beck Depression Inventory; BKMI- The Blatt–Kupperman Index

Table S4. The social support according of ISSB of the respondents depending on medical variables

Variables		ISSB							
		emotional support		information support		instrumental support		appraisal support	
		**M**	**SD**	**M**	**SD**	**M**	**SD**	**M**	**SD**
**Menopausal status**	Perimenopasal women (n=98)	31.67	7.56	45.63	11.24	57.86	11.97	16.74	5.09
	Postmenopausal women (n=120)	32.52	8.59	47.7	11.68	59.63	9.82	18	5.14
	p*	0.38		0.074		0.416		0.06	
**Get over COVID-19**	Yes (n=90)	31.09	8.52	45.51	11.79	57.3	11.83	16.74	5.49
	No (n=128)	32.88	7.81	47.66	11.26	59.91	10.01	16.74	5.49
	p*	0.102		0.138		0.151		0.115	

M—mean; SD—standard deviation. p—significance level. * Kruskal-Wallis test ^ & Mann-Whitney test, p—significance level, ISSB- The Inventory of Social Supportive Behaviors
